# Experiences and Attitudes on Early Identification Practices of Autism: A Preliminary Survey of Pediatric Professionals in Ecuador

**DOI:** 10.3390/children9020123

**Published:** 2022-01-18

**Authors:** Paulina Buffle, Alfredo Naranjo, Edouard Gentaz, Giacomo Vivanti

**Affiliations:** 1 Department of Psychology, Faculty of Psychology and Educational Sciences, University of Geneva, 1211 Geneva, Switzerland; edouard.gentaz@unige.ch; 2 School of Pediatrics, Faculty of Medicine, Pontificia Universidad Católica de Quito, Quito 170143, Ecuador; janaranjo@puce.edu.ec; 3 Swiss Center for Affective Sciences, University of Geneva, 1202 Geneva, Switzerland; 4 A.J. Drexel Autism Institute, Drexel University, Philadelphia, PA 19104, USA; gv89@drexel.edu

**Keywords:** autism spectrum disorder, child development, pediatrics, family medicine, autism screening, barriers, Ecuador

## Abstract

Early identification of children with autism is necessary to support their social and communicative skills and cognitive, verbal, and adaptive development. Researchers have identified several barriers to early diagnosis. Data collected in low- and middle-income contexts—where the vast majority of children in the world live—is scarce. In Ecuador, as in many other countries, estimates are lower compared to the global prevalence. Health authorities estimate a prevalence of 0.28% (0.18%–0.41%) in children aged five years old or less. Based on the hypothesis that, as in many parts of the world, children in Ecuador are not routinely screened and that this situation may result from poor recognition of this condition, our objective was to identify potential obstacles to case identification in pediatric settings. Several barriers, consistent with those identified in other countries, were reported by 153 participants on a survey, including lack of time, lack of resources to refer, fear of unnecessarily alarming families, and lack of information to guide families with concerns. The vast majority of participants are aware of the need for a screening tool for autism detection but report a lack of knowledge of its formal application. Most of the barriers reported in this study could be overcome by educational programs tailored to professional needs in order to support the well-being of children with autism and their families.

## 1. Introduction

Autism Spectrum Disorder (ASD) is defined by a heterogeneous group of behavioral symptoms that emerge over the first years of life and is characterized by difficulties in social communication, repetitive behaviors, and restricted interests [1]. Recent studies have reported a prevalence of at least one per cent [2,3,4]. Estimates can vary considerably between studies, regions of the world, and over time, due to differences in sampling techniques, participant’s symptoms’ severity, inclusion criteria, and the degree of qualification of professionals who evaluate the cases. In Latin America, the first epidemiological findings have indicated lower estimates compared to most high-income countries: Argentina (0.13%) [5], Brazil (0.27%) [6], Mexico (0.87%) [7], Venezuela (0.17%) [8].

Etiology and developmental paths have been intensely studied in the last years, revealing very diverse behavioral, cognitive, and neural individuals’ developmental trajectories. More than 100 genes have been related to the risk of ASD [9], but environmental factors may play an equally prominent role [10]. ASD is also characterized by a range of co-occurring conditions, such as epilepsy, which will affect at least 20–30% of individuals with ASD at one point during their lives [11]. Similar percentages of these individuals will be affected by gastrointestinal conditions [12] or sleep problems [13].

Behavioral markers characteristic of this condition can be visible from the first year of life [14], and a reliable diagnosis is possible from 24 months of age [15,16,17]. Identifying children with ASD before the age of 3 years is important, as the age at which a child with ASD begins the intervention is considered a critical indicator of their development [18]. A late diagnosis may hinder the implementation of adequate support and intervention strategies and overlook the critical period of early development [19]. A late diagnosis can also delay the diagnosis of other co-occurring conditions. 

Developmental surveillance of young children in pediatric settings is recommended by the American Academy of Pediatrics (AAP) [20]. It comprises questioning caregivers about their developmental concerns, children’s informal observation, and symptom monitoring in routine health supervision. However, this practice could be insufficient by itself to detect ASD because parents may not report concerns unless specifically asked or because some children may not demonstrate characteristic symptoms during brief visits [21]. The AAP also recommends using a standardized developmental tool in all children at specific intervals (i.e., at the 9-, 18-, and 24- or 30-month visits) regardless of concerns or risks identified during the surveillance process. However, tools used to detect language, cognitive, and motor development delays may not be sensitive to symptoms associated with ASD [22,23]. Thus, using a specific ASD tool is advised, whether parents raise concerns spontaneously or after the clinical observation [20]. The latest AAP recommendations also suggest the use of valid assessment tools, with adequate compensation for health personnel for the time required for administration, scoring, and advice related to screening [24]. 

Despite the efforts to support early identification of children with ASD, several studies have found that the mean age of diagnosis in high resources settings ranges from 50 to 60 months [25,26,27,28]. Various factors have been associated with an earlier age of diagnosis, such as a higher degree of symptoms’ severity, the presence of language delay, or a higher socioeconomic status [29]. In low and middle-income settings, a certain number of studies have reported the mean age of diagnosis ranging between 45 and 72 months, as is the case in Venezuela [30], Colombia [31], Mexico [32], Nigeria [33] and India [34,35,36]. However, some of these studies have primarily only included children with severe autism symptoms, a characteristic that has been associated with an earlier diagnosis [37]. 

Specific family characteristics can also play a role in delaying a diagnosis. For example, studies performed in the US have reported that Hispanic parents are less likely to endorse causal explanations of ASD that lead to help-seeking actions than non-Hispanic white American parents [38,39]. Specific characteristics of health systems, such as the lack of availability of diagnostic services or a difficult access to them [40], and a scarcity of trained professionals [41] can also have an impact. The clinical abilities a professional may have could also impact the age of diagnosis. For example, a US study indicated that parents reported concerns related to ASD to professionals earlier than parents of children with other developmental conditions in their sample. However, children with ASD were diagnosed at a significantly older age than the children with other developmental concerns and up to three years after first addressing their first concerns [42]. This study also noted that the parents of the children with ASD were more likely to receive reassuring or passive responses, such as “the child will grow out of it”, “it is too early to tell”, or “nothing is wrong” than the parents of the children with other developmental conditions. Reasons explaining why professionals do not adopt active responses, such as referring a child for an assessment, are not clear, but could be related to the underestimation of concerns that parents raise [43], or alternatively, that professionals share parents’ concerns but cannot screen or refer to developmental specialists [44]. Some other factors have been identified as barriers to diagnosis in pediatric settings. They include a lack of self-confidence to identify signs and symptoms [41,45], perceptions of ASD as a poorly defined condition [45,46], and lack of familiarity with ASD [47].

An essential issue for early diagnosis of autism is the extent to which pediatric professionals can be involved. Pediatric professionals are in a privileged position to respond to the challenges of early identification and the management of comorbidities. They often are the first point of contact for families with young children, and they are suited to be receptive to parental concerns [46]. Furthermore, pediatric professionals can formulate practical recommendations related to the implications of a diagnosis and existing treatments and supports [22]. Similarly, family physicians can play a significant role in providing care to children in rural and smaller communities, and particularly in low- and middle-income countries [48]. However, little is known about screening practices in pediatric settings, the perceptions, and experiences of ASD identification practices, and the type of challenges they face, particularly in low to middle income countries.

Ecuador is an upper-middle-income country with a population under 18 years of age estimated at 6,298,788 [49]. Aiming to ensure the prevention, detection, treatment, and rehabilitation of disabilities, the Ecuadorian parliament approved a law on disability in 2012 [50]. That same year, autism-related conditions were recognized as a disability, a status that aimed to provide specific rights for people with a diagnosis. Among the most recent efforts, the publication of an official guide provides information on identification and treatment of children younger than five years old in clinical practices [51]. The public health system has two pediatric hospitals in two major cities, and community centers throughout the county. The fields of neuro-pediatrics and neuro-psychiatry depend on professionals trained in other countries who return to work in Ecuador. Training in general pediatrics is available in the two largest cities. There is limited knowledge on the characteristics of individuals with a diagnosis of ASD in Ecuador. Preliminary evidence based on responses provided by school principals in Quito estimated that the attendance of children diagnosed with ASD corresponds to 0.11% among 453 students from 161 regular schools [52]. According to the health authorities, the prevalence in 2015 was 0.28% (0.18%–0.41%) in children under five years of age [51]. To our knowledge, the reason why these estimates are notably lower than international estimates has not been studied. However, as in many parts of the world, children in Ecuador may not be routinely screened for ASD, and this situation may result from barriers and challenges that pediatric professionals face in their practice.

Our main objectives were to describe (1) the factors influencing clinical and formal ASD identification and screen practices in pediatric settings, (2) the sense of self-efficiency and the perception of knowledge possessed concerning clinical identification and formal screening, (3) self-perceived needs related to ASD education, and (4) their own experience of case detection during their years of practice.

## 2. Materials and Methods

### 2.1. Participants and Procedure

The Ecuadorian Society of Pediatrics contributed to recruitment by sending a letter of invitation to members who were able to be contacted by email (*n* = 280). Fifty-three participants responded to the survey online. An additional 106 participants, family medicine practitioners, pediatrics residents, and family medicine residents were invited to participate via their medical school. Twelve family medicine practitioners, 81 residents in pediatrics, and 3 residents in family medicine accepted to complete the survey, and returned a paper version of the survey to a research assistant. Overall, 20.3% of pediatricians and 90.5% of residents in pediatric, in family medicine and family doctors, working in public and private contexts, or in both, and from different regions of Ecuador, completed the questionnaire (Table 1). 

### 2.2. Survey Instrument

We developed a questionnaire based on the literature. We adapted it to the Ecuadorian context, considering the opinion of local physicians with experience in general pediatrics and research. We included factors previously described as influencing routine screening practices, such as lack of time to screen, and factors related to the perception of formal screening application, such as the believe that formal screening is a “lengthy procedure.” We also included elements about the sense of self-efficacy, defined as beliefs individuals hold about their ability to implement their knowledge and skills in a particular task, which could be considered a mediator of clinical behavior [53], for example, “Do you feel comfortable talking about early signs with parents?” We were also interested in the participants’ perception about the knowledge they possess on ASD identification, for example, “Do you think they you possess enough knowledge to identify autism in children clinically?” and their awareness about resources for ASD in their communities. Finally, we were interested in the participants’ opinions about their need for training for example, “Do you feel you could benefit from consistent training on ASD identification practices?” and their experience identifying ASD cases during their years of practice [45,53,54,55]. The survey included sixteen Likert scale and yes-no questions [56]. Likert items were rated 1 (strongly agree), 2 (agree), 3 (disagree), 4 (strongly disagree) and were coded, ensuring that scores reflected adherence to the propositions. For analysis purposes we simplified the 4-point Likert variable into a dichotomous variable (agree 1,0). Although dichotomization implies the loss of some of the variance it allowed more visibility to infer the items that could be considered problematic for daily screening routine.

## 3. Results

### 3.1. Factors Influencing Routine Screening Practices

The most common factors perceived as negatively influencing routine identification practices were lack of time (69.3%), lack of resources to refer (69.3%), fear of unnecessarily alarming families if using a formal screening (58.8%).

### 3.2. Perception of Formal Screening

The vast majority of participants (92.2%) were aware of the need for an autism-specific screening tool and knew where to find one (77.1%). However, a substantial proportion of participants endorsed barriers such as not knowing when formal screening should be applied (34%), how to proceed (43.1%), or that formal ASD screening is a lengthy procedure (59.5%). 

### 3.3. Sense of Self-Efficacy

A substantial proportion of participants reported having a confident knowledge about early signs (66%), feeling comfortable with their ability to refer positive cases for evaluation (49.7%), and feeling comfortable talking about early signs with parents (41.2%).

### 3.4. Self-Perceived Knowledge Possessed

A majority of participants reported a lack of previous education on ASD clinical identification (67.3%), and the same proportion (67.3%) think they do not have enough knowledge to identify autism in children clinically. Furthermore, 26.1% do not know the type of professional they can refer a child for a follow-up, and (44.4%) lack information to guide families who are having concerns about autism. A positive piece of information is that only a very small proportion of participants (1.3%) believe autism has no treatment. 

### 3.5. Self-Perceived Needs of Training

Two thirds of participants reported needing consistent training on ASD identification practices, and 98% were interested in receiving continuing education on ASD.

### 3.6. Identification Experiences

In our sample 144 participants reported having at least two years of experience in a pediatric setting. The majority of these participants reported not having identified ASD cases (62.1%). Among the 37.9% who reported having detected positive cases, a majority (93.1%) report the identification of 1 to 6 cases during their years of practice.

## 4. Discussion

This study aimed to describe experiences and attitudes in pediatric settings regarding ASD identification of young children in Ecuador. As they are in contact with families of young children, pediatricians and family physicians can play an essential role in the early identification of ASD. Furthermore, these professionals can be receptive to parents’ concerns, and provide recommendations related to diagnosis, treatments, and existing supports [22]. This role is of particular relevance for children living in low- and middle-income countries, where ASD specialists may be scarce [34]. Nevertheless, universal screening for autism in young children in general pediatric settings remains a challenge in many countries worldwide.

Using a questionnaire developed on the basis of past research and adapted to an Ecuadorian context, we explored a set of factors that can negatively influence identification practices. We found that, consistent with past studies, lack of time was endorsed as a prime barrier [54,55]. Indeed, a significant challenge for clinicians in pediatric settings is the balance between implementing preventive health recommendations, focusing on parental concerns, and being aware of time constraints [45]. Nevertheless, a relevant element that would need to be observed in future studies is to know whether screening practices are adequately funded, as this aspect has also been reported to be a barrier [29], particularly in contexts where the additional time dedicated to screening is not reimbursed or is included within the preventive care visit [45].

In this study we also found that an equally large proportion of participants endorsed the lack of resources to refer a child. This finding is consistent with a recent study on families’ needs in Latin America that points to a lack of specialized structures and services as important barriers [57]. This result suggests that in Ecuador, the decision to use a screening tool may be in some cases related to the belief that “what good will it do to screen if we can’t provide services?” as effectively described by Fenikilé and col. (2015) [45]. These results also suggest that in Ecuador, as in many other countries, the barriers to detection may be equally related to the challenges in physicians’ practices, and to limited resources provided by the health systems. The availability of resources to respond to the needs of early identification have not, to our knowledge, been studied. However, the availability of resources for young children is necessary in order to access early intervention services and support for their families. 

Regarding formal autism-specific screening tools, they are mainly perceived as a long process by our participants, and the identification of an instrument adapted to the age and situation of a child seems to represents a challenge for a substantial proportion of them. Several studies have reported an underuse of ASD formal screening instruments, and this situation has been related to time constraints or to a lack of familiarity with the instruments [45,47,54,55]. Furthermore, its application has been described as dependent on parents transmitting their concerns to clinicians [58] and, in the case of family doctors, its application could be dependent on the result of their own observations [45]. In our sample, nevertheless, almost all participants endorsed the importance of using a formal tool during early identification routines. Together, these findings suggest that concise and specific training aiming to clarify details about the selection and application of a screening instrument can allow seizing the opportunities offered by a positive attitude towards the instruments.

The study of self-perceived knowledge and the sense of self-efficacy indicated that two-thirds of participants possess enough knowledge about the type of professional to whom they could refer a child for follow-up. Moreover, a considerable proportion of participants reported confident knowledge about ASD early signs (66%) and half of the participants feel comfortable with their ability to refer positive cases for evaluation to a specialist. However, these results could contradict the fact that most of our participants (67.3%) believe they do not have enough knowledge to clinically identify autism in children. This gap between the perception of possessing a theoretical knowledge and the self-perceived ability to detect signs clinically could be explained by the exposition of physicians to information about early signs, without the opportunities to develop clinical skills. In any case, these are aspects that would need further investigation to be clarified. Our results also indicate that a significant proportion of the participants feel comfortable talking about a child’s ASD early signs with his family (41.2%), but more than half of the participants endorsed the hesitation of unnecessarily alarming families if using a formal evaluation (58.8%). Furthermore, an important proportion of participants believe they lack information to guide families who have concerns about ASD. This contradictory information could be partially explained by the difficulty to discuss suspected autism with parents [45,54]. More research would be necessary, however, to identify specific barriers within the interaction with families and to understand the impact on ASD identification and case management. Finally, in our sample a minimal proportion of participants believes that autism has no treatment, suggesting that with available resources physicians would implement screening recommendations.

Experiences of ASD identification were particularly limited in our sample. The majority of pediatricians and family physicians who reported at least two years of practice in a pediatric context had never detected ASD cases and among the professionals who did have experience in detection, a majority (93.1%) had identified between 1 and 6 cases during their years of practice. These figures confirm the low official estimates [51]. Finally, concerning the perception of their own needs for training, two-thirds of the participants reported not having had specific education on ASD during their professional training, and almost all were interested in receiving continuing education on the subject.

Together, these findings point out the need to respond to the professionals’ specific gaps in knowledge and clinical exposure to young children with ASD and their families, in order to expand their skills and sense of self-efficacy [53]. The integration of specific theoretical and clinical training within academic curricula and continuous education may be a priority. A thoughtful next step would be to examine current screening practices in urban and rural settings, and to observe the adaptability of screening instruments to the local population [59], including poorly studied groups, such as women or minorities. Finally, it would be important to examine the demographic correlates in order to identify elements that predict detection routines in private practices, public hospitals, and rural community centers, while carefully evaluating new initiatives to increase the chance that an effective ASD screening strategy will be integrated into general pediatric practice.

This study was limited by aspects specific to survey designs. Firstly, to study the ASD barriers in pediatric settings we chose to survey family physicians and pediatricians, as the former are possibly more present in rural communities. However, pediatricians may have more children in their practices and therefore have more established views on identification and ASD practices and are also more likely to follow practice guidelines on ASD. Secondly, although we aimed for a national sampling frame, and 13 of the 24 provinces were represented, a substantial proportion of the participants responded from the capital and its suburban area, so these results are not indicative of the situation in rural areas, which generally face more significant barriers and may employ other categories of health professionals. Furthermore, although we identified licensed general pediatricians, we had no information on their type of practice or whether they were active. Therefore, we could not determine if part of the non-respondents were professionals not eligible for this study because they were practicing in a pediatric subspecialty and did not answer the survey for this reason. Henceforth, we were unable to determine whether there was any systematic difference between respondents and non-respondents that may have biased these findings. Additionally, participants who responded may have been more likely to be interested in autism, which could impact the results, particularly on the perception of needing consistent training on ASD identification practices and their interest in receiving continuing education on ASD. 

## 5. Conclusions

This study investigated beliefs, attitudes, and experiences about ASD identification practices in pediatric settings in Ecuador. To the best of our knowledge, this is the first study providing details to understand the needs and challenges of pediatric professionals in a Latin American country. The opinions expressed in this survey provide important perspectives on factors that have received little attention, but that are nevertheless essential to improving identification of young children needing support for their social, cognitive, and emotional development. In opposition to systemic barriers, such as family characteristics, most of the professionals-related factors reported in this study could be overcome through a cost-effective training and support for the development of clinical skills that can enhance a sense of self-efficacy in pediatrics and in family medicine.

## Figures and Tables

**Table 1:** Demographics.

Variables	*n* = 153
Gender	
Male	41
Female	112
Age	
Mean	38.8
Range	22–74
Profession	
Pediatricians	57
Family Doctors	12
Resident in Pediatrics	81
Residents in Family Medicine	3

## Data Availability

The data that support the findings of this study are available on request from the corresponding author, [PB]. The data are not publicly available due to restrictions on information that could compromise the privacy of research participants.
